# Optimization of N-P-K Nutrient Ratios for Three Leafy Vegetables Using Response Surface Methodology and Principal Component Analysis

**DOI:** 10.3390/plants14233681

**Published:** 2025-12-03

**Authors:** Ruiping Yang, Hao Su, Jiangshan Lai, Yu Sheng, Yu Shen

**Affiliations:** 1 Jiangsu Provincial Key Laboratory of Environmental Engineering, Jiangsu Provincial Academy of Environmental Science, Nanjing 210037, China; 2 Co-Innovation Center for the Sustainable Forestry in Southern China, College of Ecology and Environment, Nanjing Forestry University, Nanjing 210037, China; 3 Jiangsu Key Laboratory for Bioresources of Saline Soils, Jiangsu Synthetic Innovation Center for Coastal Bio-Agriculture, School of Wetlands, Yancheng Teachers University, Yancheng 224007, China; 4 College of Resources and Environmental Sciences, Nanjing Agricultural University, Nanjing 210095, China

**Keywords:** *Spinacia oleracea*, *Brassica rapa*, fertilizer optimization, response surface methodology, precision horticulture, principal component analysis

## Abstract

This study determined the optimal nitrogen–phosphorus–potassium (N-P-K) ratios for maximizing growth performance in spinach (*Spinacia oleracea*), bok choy (*Brassica rapa* subsp. *chinensis*), and Chinese cabbage (*Brassica rapa* pekinensis). A response surface methodology experiment with 15 N-P-K treatments (0–1.5 g/L per nutrient) was conducted under controlled conditions. Growth parameters including plant height, biomass, leaf area, and root development were measured after four weeks and analyzed using principal component analysis and Pearson correlation analysis. Optimal ratios were species-specific: spinach achieved maximum performance with N-P-K = 2-0-2 (13.15 g fresh weight, 13.88 g total biomass), bok choy with N-P-K = 0-2-2 (2631.31 mm^2^ leaf area, 4.42 mm stem diameter), and Chinese cabbage with N-P-K = 2-0-2 (14.14 cm height, 9883.44 mm^2^ leaf area). High nitrogen levels were negatively correlated with root development across all species (r = −0.531 to −0.690, *p* < 0.05). These findings demonstrate that species-specific nutrient management strategies are essential for optimal leafy vegetable production. Balanced N-P-K ratios prevent nutrient toxicity while maximizing growth, providing evidence-based guidelines for precision fertilization in controlled environment agriculture.

## 1. Introduction

Fertilizers play a crucial yet potentially hazardous role in global vegetable production [1]. While nitrogen (N), phosphorus (P) and potassium (K) enable crops to achieve robust photosynthesis, growth and yields [2], excessive synthetic fertilizer use threatens groundwater contamination, eutrophication and exacerbated climate change [3,4]. Tailored nutrient management plans aligned to soil types, cultivated crops and regional climate are therefore imperative to balance agricultural productivity with ecological sustainability [5,6]. Leafy vegetables are essential dietary components worldwide, providing high nutritional value and rich in various vitamins, minerals, and dietary fibers, which play a vital role in maintaining human health [7]. China has a long history of leafy vegetable production and consumption. With rising living standards and increased awareness of healthy eating, the market demand for leafy vegetables has been increasing, making them essential economic crops in facility agriculture [8]. However, the growth of leafy vegetables is readily influenced by environmental conditions and cultivation management practices, especially the supply of key mineral elements such as N, P, and K [9,10].

As rising populations heighten nutritional requirements amid limited arable lands, enhancing efficiency of vegetable cultivation is critical [11,12]. Vegetables provide essential dietary vitamins, minerals, and fiber [13]. However, over-fertilization continues to drive nutrient runoff, leaching, and gaseous emissions while yielding marginal productivity gains [14]. Optimizing N-P-K nutrient solution ratios to improve growth performance and quality while achieving balanced fertilization has become a pressing issue in protected vegetable cultivation.

Although previous studies have extensively investigated the effects of different N-P-K levels on the growth of leafy vegetables, there are still some limitations. Experimental design and analytical method constraints often make it difficult to accurately evaluate interactive N-P-K effects [15,16]. On the other hand, the differences in leafy vegetable varieties, substrate types, and environmental conditions make it difficult to unify N-P-K requirements [17]. Additionally, variations in environmental conditions and cultivation modes also affect fertilizer utilization efficiency, leading to a lack of universality in research conclusions [18]. Comparative studies using advanced analytical methods are therefore needed to determine optimal N-P-K ratios for leafy vegetables. The optimal use of fertilizers in vegetable crop production has long been an issue of great importance [19]. Balancing crop yield, farm profitability and ecological impact remains a key challenge for future agriculture.

The present study used spinach, bok choy, and Chinese cabbage as experimental materials and employ principal component analysis to systematically investigate the effects of different NPK nutrient solution ratios on their growth characteristics and biomass, and identify the optimal nitrogen, phosphorus, and potassium combinations. The findings provide theoretical foundations and practical guidance for the facility cultivation of leafy vegetables. Our main scientific hypotheses include: (1) N-P-K ratios have significant effects on the growth of different leafy vegetables, and the direction and magnitude of the effects vary with vegetable species; (2) there is an optimal N-P-K ratio that can maximize growth and biomass accumulation, and this ratio varies with vegetable species; (3) principal component analysis can be used to select key N-P-K components from multiple indicators and achieve comprehensive evaluation. This study aims to reveal relationships between N-P-K nutrition and leafy vegetable development, providing scientific, precise, and environmentally sound fertilization guidance with significant academic value and practical implications for achieving high-quality, efficient, and sustainable protected vegetable production.

## 2. Materials and Methods

### 2.1. Plant Materials and Growth Conditions

The seeds of spinach (*Spinacia oleracea*), bok choy (*Brassica rapa* subsp. *chinensis*), and Chinese cabbage (*Brassica rapa* pekinensis) used in this study were commercial ‘Suzhou Qing’ cultivar seeds obtained from Xingyun Vegetable Seed Breeding Center, Qing County, China. These three leafy vegetables are among the major leafy vegetables cultivated in China and throughout East Asia [20,21]. The Chinese cabbage cultivar used was a loose-leaf type suitable for short-duration cultivation, appropriate for the four-week experimental period [22,23]. The germination process took place in a seedling box maintained at a temperature of 20/25 °C (night/day) and a humidity level of 50%, utilizing ZLC-100D equipment from Shuolian Equipment Co., Ltd. (Hangzhou, China). After four weeks, when the seedlings had developed three true leaves, we carefully selected uniform seedlings for subsequent experiments. For spinach, which is sensitive to transplanting stress, seedlings were transplanted with minimal root disturbance and maintained under stable environmental conditions during the establishment period to reduce transplanting shock.

The experiment employed a completely randomized design with three factors (nitrogen, phosphorus, and potassium) at four levels each (0, 0.5, 1.0, and 1.5 g/L), resulting in 15 treatment combinations determined by response surface methodology (Table S1). Each treatment was replicated five times, with three plants per replicate, yielding 15 plants per treatment and a total of 225 experimental units across all treatments. For the experiments, groups of three seedlings were transplanted into plastic pots measuring 10 × 7 × 8.5 cm, each equipped with a 1 cm diameter drainage hole at the bottom. To prevent soil leakage, a 20-mesh insect-proof net (5 × 5 cm) was placed at the bottom of each pot.

The substrate was a commercially available peat-based seedling medium (Shaanxi Yangling Yufeng Seed Industry Co., Ltd., Xianyang, China) with particle size distribution of <0.5 mm (45%), 0.5–1.0 mm (35%), and >1.0 mm (20%), bulk density of 0.35–0.45 g/cm^3^, and water-holding capacity of 65–75%. The substrate had a pH of 6.5–7.0, electrical conductivity of 0.8–1.2 mS/cm, organic matter content >60%, and total porosity of 85–90%. Initial nutrient content before fertilizer application was as follows: available nitrogen 80–120 mg/kg, available phosphorus 40–60 mg/kg, and available potassium 100–150 mg/kg. Each pot (10 × 7 × 8.5 cm) contained approximately 400 mL of substrate, providing approximately 133 mL per plant.

The four-week cultivation period was selected to evaluate vegetative growth response during the rapid growth phase, corresponding to the baby leaf to young plant stage (5–8 true leaves). This growth stage represents a critical period for nutrient optimization and is economically relevant for fast-turnover leafy vegetable production in controlled environment agriculture and greenhouse production systems [24].

### 2.2. Fertilizer Treatments and Application

The nitrogen–phosphorus–potassium treatments were supplied using commercially available analytical grade fertilizers: urea (CO(NH_2_)_2_, 46% N) as the nitrogen source, monopotassium phosphate (KH_2_PO_4_, 52% P_2_O_5_, equivalent to 22.8% P) as the phosphorus source, and potassium chloride (KCl, 60% K_2_O, equivalent to 49.8% K) as the potassium source. The model incorporated the following variables, Unit 0 = 0 g/L (control), Unit 1 = 0.5 g/L, Unit 2 = 1.0 g/L, and Unit 3 = 1.5 g/L for each nutrient element. For example, Treatment T6 (N-P-K = 2-2-2) contained 1.0 g/L N (supplied as 2.17 g/L urea), 1.0 g/L P (supplied as 4.39 g/L KH_2_PO_4_), and 1.0 g/L K (supplied as 2.01 g/L KCl plus potassium from KH_2_PO_4_).

Fertilizer solutions were prepared fresh weekly using deionized water and applied to seedlings at seven-day intervals, beginning one week after transplanting. Each pot received 100 mL of the designated nutrient solution per application, for a total of four applications over the four-week experimental period. The control treatment (T1, N-P-K = 0-0-0) received only 100 mL of deionized water at each application time.

### 2.3. Screening Experiment and Measurements

A response surface model (utilizing Design Expert 9 from Stat-Ease, Minneapolis, MN, USA) was employed to identify the optimal N-P-K solution for promoting the growth of spinach, bok choy, and Chinese cabbage, as outlined in Table S1 [25]. Throughout the experiment, measurements of plant heights and stem diameters were taken weekly. After four weeks, the plants underwent harvesting. From each treatment, nine seedlings were randomly chosen. The above- and belowground parts were separated for measurements.

Plant height and stem diameter were measured weekly throughout the experiment. Stem diameter was measured at the base of the stem (approximately 1 cm above the substrate surface) using digital calipers (precision ±0.01 mm). Root length was measured from the base of the stem to the tip of the main root after carefully washing the roots with tap water. Leaf area was determined using the grid method on fresh leaves [26]. Fresh weights of above- and belowground parts were measured immediately after separation using an analytical balance (precision ±0.001 g). For dry weight determination, plant samples were oven-dried at 105 °C for 30 min to deactivate enzymes, followed by 80 °C for 24 h until constant weight was achieved [27]. All measurements were performed following standard protocols for vegetable growth analysis [28].

### 2.4. Statistical Analysis

For data containing replicates, variance analysis and principal component analysis were carried out using the R Project (v4.3.2). The statistical analysis and the Pearson’s correlation analysis of the data were conducted using SPSS 21.0 statistical software (IBM Corp., Armonk, NY, USA) [29]. Figures were generated using Origin Lab 9.0 (Northampton, MA, USA). Means were compared using the least significant difference test and Duncan’s new multiple range test. In this study, statistical significance was set at the level of *p* < 0.05.

## 3. Results and Discussion

### 3.1. Spinach N-P-K Formula

**Figure 1:**
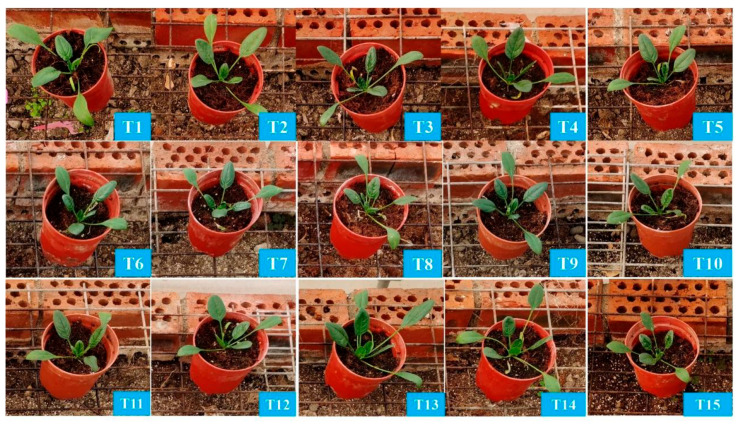
The phenotype of spinach (*Spinacia oleracea*) after four-week treatments. (**T1**): N-P-K = 0-0-0, (**T2**): N-P-K = 0-2-2, (**T3**): N-P-K = 1-2-2, (**T4**): N-P-K = 2-0-2, (**T5**): N-P-K = 2-1-2, (**T6**): N-P-K = 2-2-2, (**T7**): N-P-K = 2-3-2, (**T8**): N-P-K = 2-2-3, (**T9**): N-P-K = 2-2-0, (**T10**): N-P-K = 2-2-1, (**T11**): N-P-K = 3-2-2, (**T12**): N-P-K = 1-3-2, (**T13**): N-P-K = 1-1-2, (**T14**): N-P-K = 1-2-1, (**T15**): N-P-K = 2-1-1, 1 unit means 0.5 g/L nutrient.

**Figure 2:**
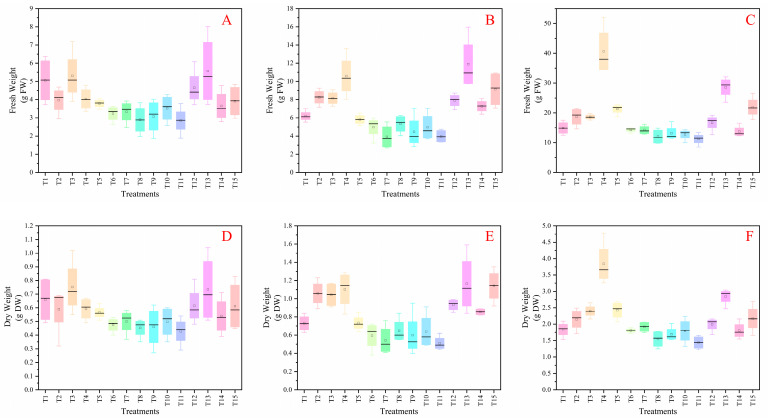
The records of total fresh weight (**A**–**C**), and the total dry weight (**D**–**F**) of spinach, bok choy and Chinese cabbage after four-week treatments, respectively. (Note, T1: N-P-K = 0-0-0, T2: N-P-K = 0-2-2, T3: N-P-K = 1-2-2, T4: N-P-K = 2-0-2, T5: N-P-K = 2-1-2, T6: N-P-K = 2-2-2, T7: N-P-K = 2-3-2, T8: N-P-K = 2-2-3, T9: N-P-K = 2-2-0, T10: N-P-K = 2-2-1, T11: N-P-K = 3-2-2, T12: N-P-K = 1-3-2, T13: N-P-K = 1-1-2, T14: N-P-K = 1-2-1, T15: N-P-K = 2-1-1; 1 unit means 0.5 g/L nutrient; values are mean ± SD; one-way ANOVA with Duncan’s test (*p* < 0.05)).

Spinach growth performance varied significantly under different nutrient treatments. Compared to the control (T1), spinach exhibited superior growth in treatments T2, T4, T13, and T14, while growth was reduced in treatments T7, T10, and T11 (Figure 1). Biomass measurements of spinach under no treatment (T1) reached above-average levels in aboveground, belowground, and total biomass fresh weight, with 9.47 g, 0.58 g, and 10.05 g, respectively (Figure 2A,D). In terms of aboveground fresh weight, spinach performed best in treatment T4 (13.15 g), followed by treatments T2, T3, T5, T12, T13, and T14. The lowest aboveground fresh weight was recorded in treatment T10 (0.79 g), followed by T9 (2.73 g) and T11 (5.19 g).

Growth indices of spinach were recorded after 4 weeks of treatments (Figure S1). Plant height was highest in T1 and T2, while T10 exhibited the lowest value (Figure S1A). Stem diameter was greatest in T2, whereas T10 had the smallest diameter (Figure S1B). Root length varied dramatically, with T1 exhibiting the longest roots, followed by T2, while T10 showed the shortest (Figure S1C). Leaf number was highest in T9, whereas T10 had the lowest value (Figure S1D). Leaf area followed a similar pattern, with T9 showing the largest area and T10 the smallest (Figure S1E). Overall, T10 consistently exhibited the poorest growth performance across all measured parameters.

**Figure 3:**
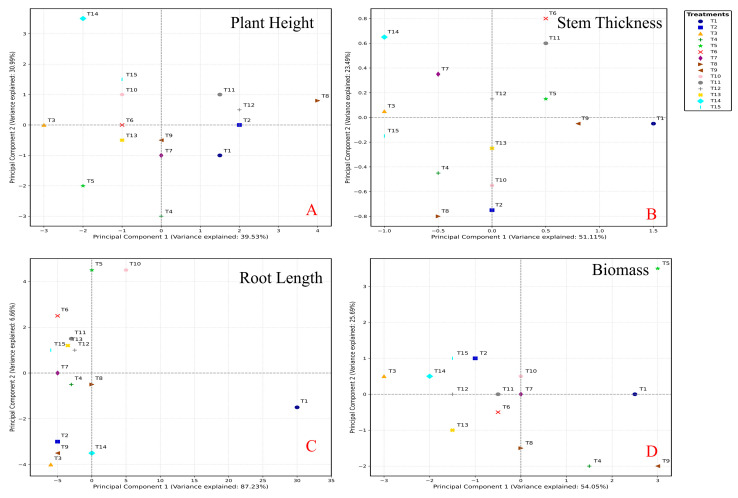
The PCA analysis of the Plant height (**A**), stem thickness (**B**), root length (**C**), and biomass (**D**) of spinach after four-week treatments, respectively. (Note, T1: N-P-K = 0-0-0, T2: N-P-K = 0-2-2, T3: N-P-K = 1-2-2, T4: N-P-K = 2-0-2, T5: N-P-K = 2-1-2, T6: N-P-K = 2-2-2, T7: N-P-K = 2-3-2, T8: N-P-K = 2-2-3, T9: N-P-K = 2-2-0, T10: N-P-K = 2-2-1, T11: N-P-K = 3-2-2, T12: N-P-K = 1-3-2, T13: N-P-K = 1-1-2, T14: N-P-K = 1-2-1, T15: N-P-K = 2-1-1; 1 unit means 0.5 g/L nutrient).

Principal component analysis (PCA) revealed that treatments T6, T9, and T10 positively contributed to biomass, while T2, T4, T5, and T13 had a negative impact on biomass growth (Figure 3A). Shoot height showed a positive correlation with treatments T9 and T10 and a negative correlation with treatments T2, T4, T8, and T15 (Figure 3B). Treatment T1 exhibited a positive correlation with root length and stem diameter, while T15 showed a negative correlation (Figure 3C). Stem diameter increases were negatively correlated with treatments T2, T4, T6, T8, T11, and T14 (Figure 3D). The results underscore significant growth improvements in spinach plants under various nutrient treatment regimens, emphasizing the crucial role of proper fertilization in optimizing the productivity of this important leafy vegetable [9,30].

The Pearson correlation coefficients (r) revealed significant relationships between N and various growth parameters of spinach, while P and K showed limited influence (Table S2). N exhibited a significant negative correlation with plant height (r = −0.531, *p* < 0.05), a highly demonstrated significant inverse correlation with stem diameter (r = −0.690, *p* < 0.01) and fresh weight (r = −0.685, *p* < 0.01), and a significant negative correlation with root length (r = −0.569, *p* < 0.05). These results suggest that increasing N levels could lead to a decrease in plant height, stem diameter, root length, and fresh weight of spinach. P showed a significant negative association with root length (r = −0.633, *p* < 0.05), indicating that higher P levels may result in shorter root lengths [31]. However, P did not have significant correlations with plant height, stem diameter, or fresh weight. K did not exhibit significant correlations with any of the measured growth parameters. The correlation coefficients among N, P, and K did not indicate strong relationships, suggesting that the interactions among these nutrients may not have a significant impact on the measured growth parameters of spinach.

### 3.2. Bok Choy N-P-K Formula

**Figure 4:**
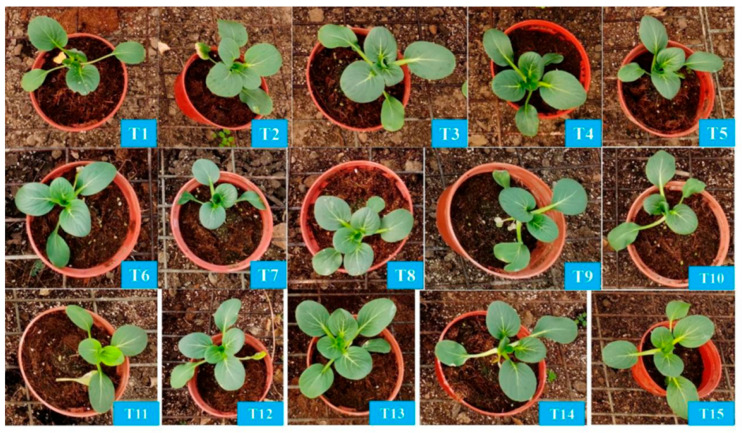
The phenotype of bok choy (*Brassica rapa* subsp. chinensis) after four-week treatments. (**T1**): N-P-K = 0-0-0, (**T2**): N-P-K = 0-2-2, (**T3**): N-P-K = 1-2-2, (**T4**): N-P-K = 2-0-2, (**T5**): N-P-K = 2-1-2, (**T6**): N-P-K = 2-2-2, (**T7**): N-P-K = 2-3-2, (**T8**): N-P-K = 2-2-3, (**T9**): N-P-K = 2-2-0, (**T10**): N-P-K = 2-2-1, (**T11**): N-P-K = 3-2-2, (**T12**): N-P-K = 1-3-2, (**T13**): N-P-K = 1-1-2, (**T14**): N-P-K = 1-2-1, (**T15**): N-P-K = 2-1-1, 1 unit means 0.5 g/L nutrient.

Bok choy growth performance varied under different nutrient treatments. Compared to the control (T1), bok choy exhibited superior growth in treatments T2, T3, T6, T8, and T13, while growth was reduced in treatments T5, T7, T10, and T11 (Figure 2B,E and Figure 4). The bok choy plant height, stem diameter, root length, number of leaves, and leaf area showed varying responses to the different nutrient treatments (Figure S2). The highest plant height was observed in T15 (11.94 cm), while the lowest was in T11 (8.38 cm). Stem diameter remained largely unaffected, with T2 having the largest (4.42 mm) and T6, T8, and T9 the smallest (3.3 mm). All treatments negatively affected root length, with T6, T7, and T9 having the shortest roots at 11.6 cm, 11.7 cm, and 11.9 cm, respectively. The number of leaves remained largely unaffected, with T15 having the highest (6) and T11 the lowest (4). Leaf area varied significantly, with T2 having the largest (2631.31 mm^2^) and T7 the smallest (1137.41 mm^2^).

**Figure 5:**
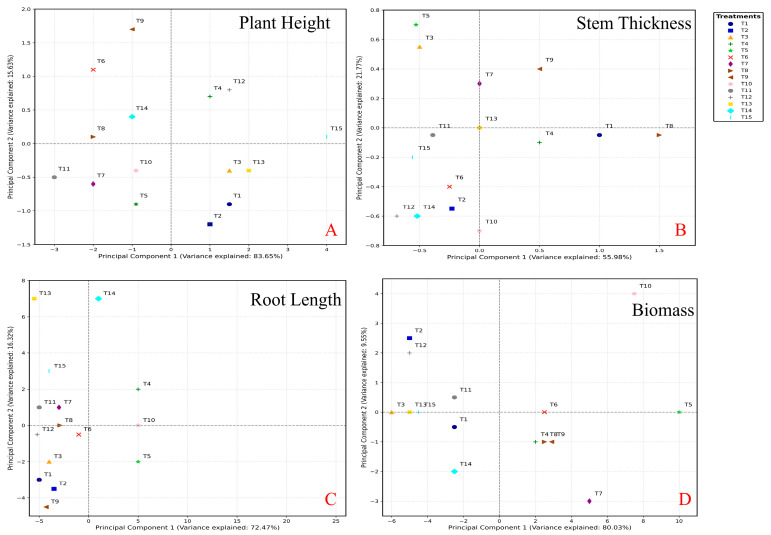
The PCA analysis of the Plant height (**A**), stem thickness (**B**), root length (**C**), and biomass (**D**) of bok choy after four-week treatments, respectively. (Note, T1: N-P-K = 0-0-0, T2: N-P-K = 0-2-2, T3: N-P-K = 1-2-2, T4: N-P-K = 2-0-2, T5: N-P-K = 2-1-2, T6: N-P-K = 2-2-2, T7: N-P-K = 2-3-2, T8: N-P-K = 2-2-3, T9: N-P-K = 2-2-0, T10: N-P-K = 2-2-1, T11: N-P-K = 3-2-2, T12: N-P-K = 1-3-2, T13: N-P-K = 1-1-2, T14: N-P-K = 1-2-1, T15: N-P-K = 2-1-1; 1 unit means 0.5 g/L nutrient).

The PCA analysis revealed distinct clustering patterns among treatments for different growth parameters (Figure 5). For plant height, PC1 and PC2 explained 83.65% and 15.63% of the total variance, respectively. T1, T2, T3, T13, and T15 positively contributed to PC1, while T6, T8, T9, and T11 had negative contributions (Figure 5A). For stem thickness, PC1 and PC2 accounted for 55.98% and 21.77% of the variance, respectively. T1, T4, T8, and T13 positively contributed to PC1, whereas T6, T10, T12, and T14 showed negative contributions (Figure 5B). Root length analysis demonstrated that PC1 and PC2 explained 72.47% and 16.32% of the variance, respectively. T4, T6, T10, and T15 had positive contributions to PC1, while T1, T2, T3, and T9 exhibited negative contributions (Figure 5C). For biomass, PC1 and PC2 explained 80.03% and 9.55% of the variance, respectively. T5, T6, and T10 positively contributed to PC1, while T2, T7, and T12 had negative contributions (Figure 5D). These results indicated that different nutrient treatments had varying effects on spinach growth parameters. The Pearson correlation coefficients (r) revealed significant relationships between N, P, and various growth parameters of bok choy, while K showed limited influence (Table S3). N exhibited a highly inverse relationship with stem diameter (r = −0.773, *p* < 0.01) and a significant negative correlation with root length (r = −0.561, *p* < 0.05), suggesting that increasing N levels could lead to a decrease in stem diameter and root length of bok choy. However, N did not show significant correlations with plant height or fresh weight. P demonstrated a significant negative correlation with root length (r = −0.578, *p* < 0.05), indicating that higher P levels may result in shorter root lengths [16,31], but did not have significant correlations with plant height, stem diameter, or fresh weight. K did not exhibit significant correlations with any of the measured growth parameters. The correlation coefficients among N, P, and K did not indicate strong relationships, suggesting that the interactions among these nutrients may not have a significant impact on the measured growth parameters of bok choy.

### 3.3. Chinese Cabbage N-P-K Formula

**Figure 6:**
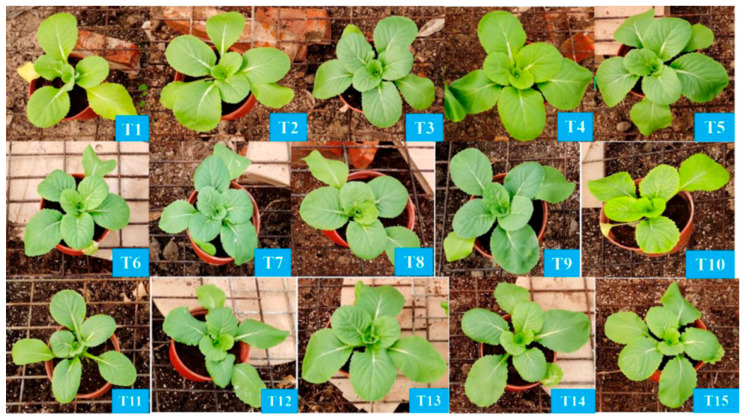
The phenotype of Chinese cabbage (*Brassica rapa* pekinensis) after four-week treatments. (**T1**): N-P-K = 0-0-0, (**T2**): N-P-K = 0-2-2, (**T3**): N-P-K = 1-2-2, (**T4**): N-P-K = 2-0-2, (**T5**): N-P-K = 2-1-2, (**T6**): N-P-K = 2-2-2, (**T7**): N-P-K = 2-3-2, (**T8**): N-P-K = 2-2-3, (**T9**): N-P-K = 2-2-0, (**T10**): N-P-K = 2-2-1, (**T11**): N-P-K = 3-2-2, (**T12**): N-P-K = 1-3-2, (**T13**): N-P-K = 1-1-2, (**T14**): N-P-K = 1-2-1, (**T15**): N-P-K = 2-1-1; 1 unit means 0.5 g/L nutrient.

The Chinese cabbage growth performance varied under different nutrient treatments. Compared to the control (T1), Chinese cabbage exhibited better growth in treatments T2, T3, T4, T5, and T13, while it grew smaller in treatments T7, T8, T10, and T12 (Figure 2C,F and Figure 6). The Chinese cabbage plant height, stem diameter, root length, number of leaves, and leaf area showed varying responses to the different nutrient treatments (Figure S3) [22,23]. The highest plant height was observed in T4 (14.14 cm), while the lowest was in T14 (11.76 cm). All treatments had a negative impact on stem diameter, with T11, T12, and T14 having the smallest stem diameters of 5.58 mm, 5.51 mm, and 5.32 mm, respectively. Similarly, all treatments negatively affected root length, with T11, T15, and T9 having the shortest roots at 11.68 cm, 12.15 cm, and 12.35 cm, respectively. The number of leaves remained largely unaffected, with T4 having the highest average of 7.5. Leaf area varied significantly, with T4 having the largest (9883.44 mm^2^) and T3 the smallest (2121.96 mm^2^).

**Figure 7:**
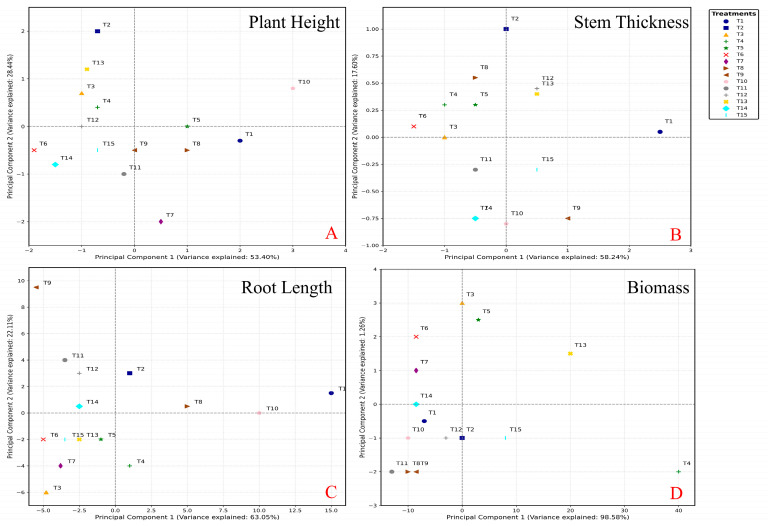
The PCA analysis of the Plant height (**A**), stem thickness (**B**), root length (**C**), and biomass (**D**) of Chinese cabbage after four-week treatments, respectively. (Note, T1: N-P-K = 0-0-0, T2: N-P-K = 0-2-2, T3: N-P-K = 1-2-2, T4: N-P-K = 2-0-2, T5: N-P-K = 2-1-2, T6: N-P-K = 2-2-2, T7: N-P-K = 2-3-2, T8: N-P-K = 2-2-3, T9: N-P-K = 2-2-0, T10: N-P-K = 2-2-1, T11: N-P-K = 3-2-2, T12: N-P-K = 1-3-2, T13: N-P-K = 1-1-2, T14: N-P-K = 1-2-1, T15: N-P-K = 2-1-1; 1 unit means 0.5 g/L nutrient).

The PCA analysis revealed distinct clustering patterns among treatments for different growth parameters (Figure 7). For plant height, PC1 and PC2 explained 53.40% and 28.44% of the total variance, respectively. T1, T8, T9, and T10 positively contributed to PC1, while T6, T14, and T15 had negative contributions (Figure 7A). For stem thickness, PC1 and PC2 accounted for 58.24% and 17.60% of the variance, respectively. T1, T11, and T15 positively contributed to PC1, whereas T7 and T14 showed negative contributions (Figure 7B). Root length analysis demonstrated that PC1 and PC2 explained 63.05% and 22.11% of the variance, respectively. T1, T8, and T10 had positive contributions to PC1, while T3, T6, T7, and T15 exhibited negative contributions (Figure 7C). For biomass, PC1 and PC2 explained 98.58% and 1.26% of the variance, respectively. T4, T5, and T13 positively contributed to PC1, while T3 and T6 had negative contributions (Figure 7D). These results indicated that different nutrient treatments had varying effects on spinach growth parameters.

The Pearson correlation coefficients (r) revealed significant relationships between P and various growth parameters of Chinese cabbage, while N and K showed limited influence (Table S4). P exhibited a highly significant negative correlation with plant height (r = −0.725, *p* < 0.01) and significant negative correlations with root length (r = −0.606, *p* < 0.05) and fresh weight (r = −0.626, *p* < 0.05), suggesting that increasing P levels could lead to a decrease in plant height, root length, and fresh weight of Chinese cabbage [31]. However, P did not show a significant correlation with stem diameter. N demonstrated a significant negative correlation with root length (r = −0.616, *p* < 0.05), indicating that higher N levels may result in shorter root lengths, but did not have significant correlations with plant height, stem diameter, or fresh weight. K did not exhibit significant correlations with any of the measured growth parameters. The correlation coefficients among N, P, and K did not indicate strong relationships, suggesting that the interactions among these nutrients may not have a significant impact on the measured growth parameters of Chinese cabbage.

### 3.4. N-P-K and Plant Growth

The PCA analysis revealed that the N-P (2-2) combination acts as a positive contributor to both biomass and shoot height increase in spinach, with P (2) also contributing to biomass, albeit to a lesser extent. These findings are supported by the crucial roles of these nutrients in plant growth and development. N serves as a building block for the growth of new stems and leaves and is an essential component of chlorophyll, which gives leaves their green color and aids in photosynthesis [32,33]. P is crucial for root development and energy transfer in plant cells [31,34], while K promotes healthy roots and aids in stress tolerance, including drought [34]. Therefore, a higher addition of N-P is likely to promote spinach leaf and shoot height growth [33].

The results underscore significant growth improvements in spinach plants under various nutrient treatment regimens, emphasizing the crucial role of proper fertilization in optimizing the productivity of this important leafy vegetable. Treatment T4, consisting of balanced NPK levels, demonstrated the most substantial gains in aboveground biomass, overall plant size, and leaf count, aligning with established research indicating synergistic effects of nitrogen, phosphorus, and potassium in facilitating robust photosynthesis and growth in leafy vegetables [1,9]. In contrast, inadequate or imbalanced nutrient levels, such as those in T10 plants subjected to the lowest fertilizer levels, hindered plant development, resulting in stunted height and biomass.

Multivariate analysis uncovered significant individual and interactive effects of N, P, and K on specific growth parameters of spinach, bok choy, and Chinese cabbage [16]. In spinach, Pearson correlation tests indicated potential inhibitory effects of high nitrogen levels on root elongation, as plants appeared to allocate resources preferentially towards aboveground organs [33]. However, optimal nutrient balancing was achieved under T4, indicating sufficient access to all three macronutrients. Similarly, PCA revealed positive associations between balanced N-P-K formulations, biomass, and photosynthesis-dependent factors such as shoot height and leaf count.

In bok choy, N exhibited a highly significant negative correlation with stem diameter and a significant negative correlation with root length, suggesting that increasing N levels could lead to a decrease in these parameters. P demonstrated an inverse correlation with root length, indicating that higher P levels may result in shorter root lengths [31]. For Chinese cabbage, P exhibited a highly significant negative correlation with plant height and significant negative correlations with root length and fresh weight, suggesting that increasing P levels could lead to a decrease in these parameters [31]. N demonstrated a significant negative correlation with root length, indicating that higher N levels may result in shorter root lengths.

These results offer valuable insights for tailoring fertilizer management to maximize productivity in the cultivation of spinach, bok choy, and Chinese cabbage. While nitrogen clearly played a dominant role in plant development, as evidenced by stunted growth under severe N-limitations, balanced nutrient rates proved superior. This aligns with previous reports highlighting synergistic benefits between nitrogen and potassium in the yields of leafy plants [30]. The findings suggest that site-specific formulations with precise tuning of N-P-K ratios can optimize the cultivation of these vegetables [35]. Further studies should explore response curves under varied soil types and ambient conditions.

### 3.5. Statistical Analysis for Screening Optimization

This study investigated the effects of different nutrient ratios on the growth parameters of spinach, bok choy, and Chinese cabbage. The results showed that the optimal nutrient ratios varied among the three vegetable species, highlighting the importance of tailoring nutrient management strategies to specific crop requirements [19].

For spinach, treatment T4 (N-P-K = 2-0-2) emerged as the best treatment, resulting in the highest aboveground fresh weight (13.15 g), total biomass (13.88 g), and overall plant size (Figure S1). This finding is consistent with previous research that emphasizes the importance of balanced nutrient ratios for optimal spinach growth [30]. The negative impact of high nitrogen levels on root elongation, as indicated by the Pearson correlation tests, suggests that excessive nutrient levels may hinder spinach root development [9].

In the case of bok choy, T2 (N-P-K = 0-2-2) showed promising results, with the largest leaf area (2631.31 mm^2^) and stem diameter (4.42 mm) (Figure S2). These findings suggest that bok choy may require higher levels of phosphorus and potassium relative to nitrogen for optimal growth [32]. The negative impact of increasing nitrogen levels on stem diameter and root length, as indicated by the Pearson correlation coefficients, suggests that excessive nitrogen may hinder bok choy development in these aspects.

For Chinese cabbage, T4 (N-P-K = 2-0-2) emerged as optimal, resulting in the highest plant height (14.14 cm), leaf number (7.5), and leaf area (9883.44 mm^2^) (Figure S3). This suggests that Chinese cabbage may require higher levels of nitrogen and potassium relative to phosphorus for optimal growth [21,22]. The negative impact of increasing phosphorus levels on plant height, root length, and fresh weight, as indicated by the Pearson correlation coefficients, indicates that Chinese cabbage may be more sensitive to excessive phosphorus levels compared to spinach and bok choy [31].

The varying responses of the three vegetable species to different nutrient ratios can be attributed to their distinct physiological and genetic characteristics. Spinach, being a leafy vegetable, may require a more balanced nutrient supply to support its rapid leaf growth and development [30]. Bok choy may have a higher demand for phosphorus and potassium due to its unique nutrient uptake and utilization mechanisms [32]. Chinese cabbage, with its larger biomass and storage capacity, may require higher levels of nitrogen and potassium to support its growth and head formation [22,23].

## 4. Conclusions

This study demonstrates that optimal nutrient ratios for spinach, bok choy, and Chinese cabbage differ, highlighting the importance of tailoring nutrient management strategies to specific crop requirements. For spinach, a balanced nutrient ratio of T4 (N-P-K = 2-0-2) resulted in the highest aboveground fresh weight, total biomass, and overall plant size. In contrast, bok choy benefited from higher phosphorus and potassium levels relative to nitrogen T2 (N-P-K = 0-2-2), as evident from the largest leaf area and stem diameter. Chinese cabbage performed best with higher nitrogen and potassium levels of T4 (N-P-K = 2-0-2), resulting in the highest plant height, leaf number, and leaf area.

However, the study also revealed that excessive nutrient levels could hinder growth and development in these vegetable species. High nitrogen levels negatively affected root elongation in spinach and stem diameter and root length in bok choy. Similarly, increasing phosphorus levels had detrimental impact on plant height, root length, and fresh weight in Chinese cabbage. These findings underscore the importance of striking a balance between providing adequate nutrients for optimal growth and avoiding excessive levels that may impede development.

To further advance understanding of nutrient management in these vegetable species, future research should focus on validating these findings in field trials across various environmental conditions. This will help determine the robustness and applicability of identified optimal nutrient ratios in real-world settings. Additionally, investigating underlying physiological and molecular mechanisms governing responses of spinach, bok choy, and Chinese cabbage to different nutrient ratios will provide valuable insights into their nutrient uptake, assimilation, and utilization processes. Such knowledge will contribute to developing targeted and sustainable nutrient management strategies that optimize growth, yield, and quality while minimizing environmental impacts.

In conclusion, this study highlights the significance of species-specific nutrient management in vegetable production. By tailoring nutrient ratios to unique requirements of spinach, bok choy, and Chinese cabbage, growers can enhance their growth and productivity. However, caution must be exercised to avoid excessive nutrient levels that may hinder root development and overall growth. Future research efforts should aim to validate these findings in diverse environmental conditions and unravel underlying mechanisms driving these growth responses, ultimately leading to development of sustainable and efficient nutrient management practices for these important vegetable crops.

## Data Availability

The datasets used and/or analyzed during the current study are available from the corresponding author upon reasonable request.

## Supplementary Materials

The following supporting information can be downloaded at: https://www.mdpi.com/article/10.3390/plants14233681/s1: Figure S1. The records of the spinach plant height (A), stem diameter (B), root length (C), number of leaves (D), and leaf area (E) after four weeks treatments, respectively; Figure S2. The records of the bok choy plant height (A), stem diameter (B), root length (C), number of leaves (D), and leaf area (E) after four weeks treatments, respectively; and Figure S3. The records of the Chinese cabbage plant height (A), stem diameter (B), root length (C), number of leaves (D), and leaf area(E) after four weeks treatments, respectively. Table S1 The NPK Response Surface Model Settings; Table S2 The matrix of Pearson correlation coefficients of NPK and growth indexes of the spinach; Table S3 The matrix of Pearson correlation coefficients of NPK and growth indexes of the bok choy; and Table S4 The matrix of Pearson correlation coefficients of NPK and growth indexes of the Chinese cabbage.
