# Supplementary material for: Optimization of N-P-K Nutrient Ratios for Three Leafy Vegetables Using Response Surface Methodology and Principal Component Analysis
Source: Plants (Basel). 2025 Dec 3;14(23):3681. doi: 10.3390/plants14233681 (PMC12694132; doi:10.3390/plants14233681)
Supplement: Supplementary file 1 [file plants-14-03681-s001.zip › plants-3953691-supplementary.pdf]

Supplemental Information

Optimization of N-P-K Nutrient Ratios for Three Leafy Vegetables Using Response Surface Methodology and Principal Component Analysis

Ruiping Yang [1,2,3], Hao Su [2], Jiangshan Lai [2], Yu Sheng [4*], Yu Shen [1,2*]

[1] Jiangsu Provincial Key Laboratory of Environmental Engineering, Jiangsu Provincial Academy of Environmental Science, Nanjing 210037, China

[2] Co-Innovation Center for the Sustainable Forestry in Southern China, College of Ecology and Environment, Nanjing Forestry University, Nanjing 210037, China

[3] Jiangsu Key Laboratory for Bioresources of Saline Soils, Jiangsu Synthetic Innovation Center for Coastal Bio-Agriculture, School of Wetlands, Yancheng Teachers University, Yancheng 224007, China

[4] College of Resources and Environmental Sciences, Nanjing Agricultural University, Nanjing 210095, China

[*] **Corresponding Authors**

Yu Shen, Ph.D.,

Email: yushen@njfu.edu.cn/sheyttmax@hotmail.com;

Yu Sheng, Ph.D.,

Email: shengyu1223@hotmail.com.

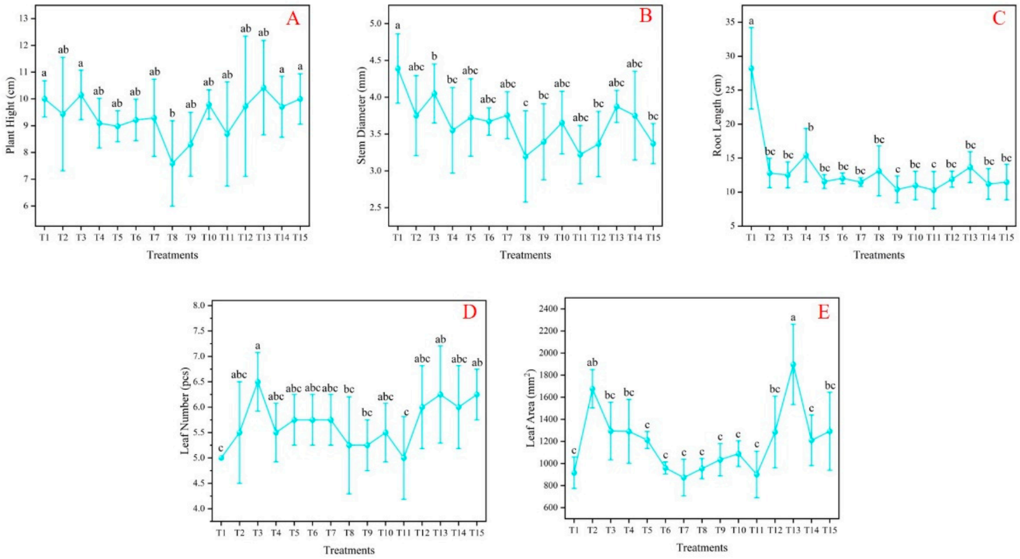

Figure S1. The records of the spinach plant height (A), stem diameter (B), root length (C), number of leaves (D), and leaf area(E) after four weeks treatments, respectively.

(Note, T1: N-P-K=0-0-0, T2: N-P-K=0-2-2, T3: N-P-K=1-2-2, T4: N-P-K=2-0-2, T5: N-P-K=2-1-2, T6: N-P-K=2-2-2, T7: N-P-K=2-3-2, T8: N-P-K=2-2-3, T9: N-P-K=2-2-0, T10: N-P-K=2-2-1, T11: N-P-K=3-2-2, T12: N-P-K=1-3-2, T13: N-P-K=1-1-2, T14: N-P-K=1-2-1, T15: N-P-K=2-1-1; 1 unit means 0.5 g/L nutrient; values are mean ± SD; one-way ANOVA with *Duncan*'s test ($p < 0.05$).

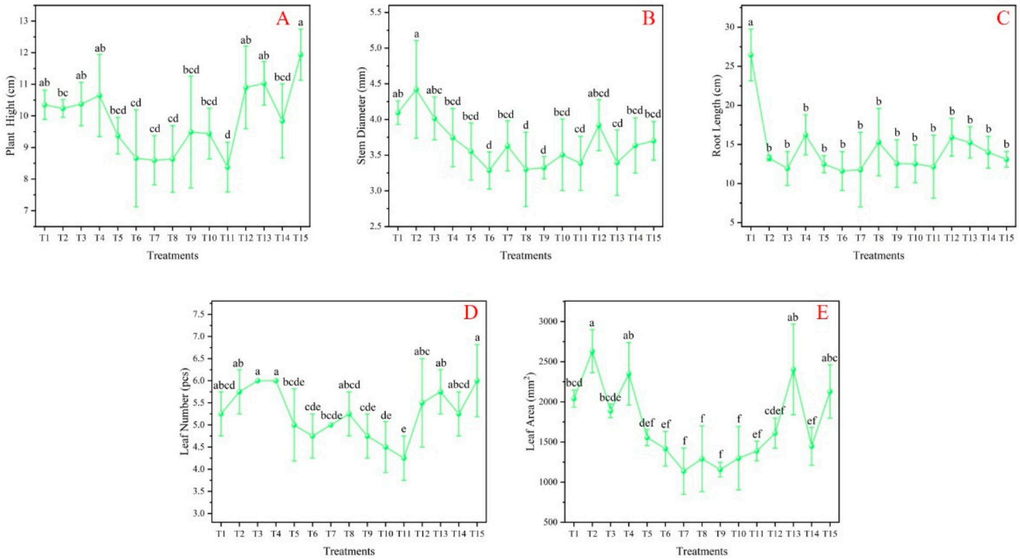

Figure S2. The records of the bok choy plant height (A), stem diameter (B), root length (C), number of leaves (D), and leaf area(E) after four weeks treatments, respectively.

(Note, T1: N-P-K=0-0-0, T2: N-P-K=0-2-2, T3: N-P-K=1-2-2, T4: N-P-K=2-0-2, T5: N-P-K=2-1-2, T6: N-P-K=2-2-2, T7: N-P-K=2-3-2, T8: N-P-K=2-2-3, T9: N-P-K=2-2-0, T10: N-P-K=2-2-1, T11: N-P-K=3-2-2, T12: N-P-K=1-3-2, T13: N-P-K=1-1-2, T14: N-P-K=1-2-1, T15: N-P-K=2-1-1; 1 unit means 0.5 g/L nutrient; values are mean ± SD; one-way ANOVA with *Duncan*'s test ($p < 0.05$).

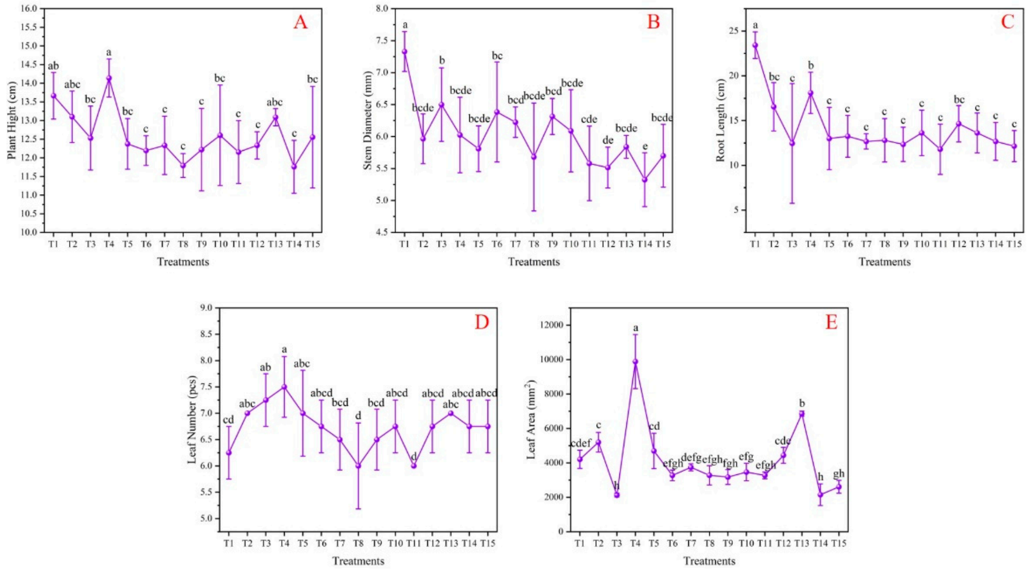

Figure S3. The records of the Chinese cabbage plant height (A), stem diameter (B), root length (C), number of leaves (D),and leaf area(E) after four weeks treatments, respectively.

(Note, T1: N-P-K=0-0-0, T2: N-P-K=0-2-2, T3: N-P-K=1-2-2, T4: N-P-K=2-0-2, T5: N-P-K=2-1-2, T6: N-P-K=2-2-2, T7: N-P-K=2-3-2, T8: N-P-K=2-2-3, T9: N-P-K=2-2-0, T10: N-P-K=2-2-1, T11: N-P-K=3-2-2, T12: N-P-K=1-3-2, T13: N-P-K=1-1-2, T14: N-P-K=1-2-1, T15: N-P-K=2-1-1; 1 unit means 0.5 g/L nutrient; values are mean ± SD; one-way ANOVA with *Duncan*'s test ($p < 0.05$).

Table S1 The NPK Response Surface Model Settings

	N	P	K
T1	0	0	0
T2	0	2	2
T3	1	2	2
T4	2	0	2
T5	2	1	2
T6	2	2	2
T7	2	3	2
T8	2	2	3
T9	2	2	0
T10	2	2	1
T11	3	2	2
T12	1	3	2
T13	1	1	2
T14	1	2	1
T15	2	1	1

Table S2 The matrix of Pearson correlation coefficients of NPK and growth indexes of the spinach.

	N	P	K
N	1		
P	0.159	1	
K	0.228	0.288	1
Plant Hight	-0.531*	-0.188	-0.271
Stem Diameter	-0.690**	-0.372	-0.342
Root Length	-0.569*	-0.633*	-0.373
Fresh Weight	-0.685**	-0.355	-0.092

Note, * Correlation is significant at the 0.05 level (2-tailed); ** Correlation is significant at the 0.01 level (2-tailed).

Table S3 The matrix of Pearson correlation coefficients of NPK and growth indexes of the bok choy.

	N	P	K
N	1		
P	0.159	1	
K	0.228	0.288	1
Plant Hight	-0.485	-0.421	-0.256
Stem Diameter	-0.773**	-0.113	-0.109
Root Length	-0.561*	-0.578*	-0.385
Fresh Weight	-0.427	-0.467	0.134

Note, * Correlation is significant at the 0.05 level (2-tailed); ** Correlation is significant at the 0.01 level (2-tailed).

Table S4 The matrix of Pearson correlation coefficients of NPK and growth indexes of the Chinese cabbage.

	N	P	K
N	1		
P	0.159	1	
K	0.228	0.288	1
Plant Hight	-0.385	-0.725**	-0.177
Stem Diameter	-0.355	-0.356	-0.454
Root Length	-0.616*	-0.606*	-0.336
Fresh Weight	-0.070	-0.626*	0.184

Note, * Correlation is significant at the 0.05 level (2-tailed); ** Correlation is significant at the 0.01 level (2-tailed).
